# The protective capability of *Hedyotis diffusa* Willd on lupus nephritis by attenuating the IL-17 expression in MRL/lpr mice

**DOI:** 10.3389/fimmu.2022.943827

**Published:** 2022-07-25

**Authors:** Ying Li, Tao Ding, Jing Chen, Jinjun Ji, Weijie Wang, Bin Ding, Weihong Ge, Yongsheng Fan, Li Xu

**Affiliations:** ^1^ School of Basic Medical Science, Zhejiang Chinese Medical University, Hangzhou, China; ^2^ Department of Rheumatology, The Second Affiliated Hospital of Zhejiang Chinese Medical University, Hangzhou, China; ^3^ School of Life Science, Zhejiang Chinese Medical University, Hangzhou, China; ^4^ School of Pharmaceutical Science, Zhejiang Chinese Medical University, Hangzhou, China

**Keywords:** *Hedyotis diffusa* Willd, lupus nephritis, network pharmacology, interleukin-17, STAT3, inflammation

## Abstract

Lupus nephritis (LN), the most severe organ manifestation of systemic lupus erythematosus (SLE), is generally treated with glucocorticoids (GC) in clinical practice, leading to drug resistance and adverse effects in the long term. Fortunately, the combination of GC and traditional Chinese medical prescriptions can attenuate the adverse effects and improve therapeutic efficiency. *Hedyotis diffusa* Willd (HDW) is one of the most commonly used herbal compounds for LN treatment, which exhibits “heat-clearing” and “detoxification” effects. However, the underlying pharmacological mechanism remains unclear. The present study identified the chemical compounds in HDW extract with UPLC-Q-TOF-MS/MS. A total of 49 components were identified in the HDW extract, and the IL-17 signaling pathway was highly enriched by network pharmacological analysis. MRL/lpr model mice, reflecting the spontaneous development of LN, were used to evaluate the protective activity and investigate the underlying mechanism of the combination treatment. The white blood cell content (WBC), including lymphocytes and neutrophils, cytokines (IL-6, MCP-1, TNF-a), and various autoantibodies (ANA, ab-dsDNA, ab-snRNP/sm) in the blood of MRL/lpr mice were significantly improved by the intragastric administration of HDW. Additionally, the expression of STAT3, IL-17, Ly6G, and MPO in the kidney and neutrophil NETosis were ameliorated with HDW treatment. The pathological and morphological analysis suggested that HDW application could reduce urinary protein levels and inflammatory cell infiltration and inhibit glomerular interstitial cell proliferation. Hence, HDW might ameliorate lupus nephritis by inhibiting IL-6 secretion and STAT3-induced IL-17 expression. The active compounds in HDW were predictively selected with computational methods. The docking affinity of asiatic acid, neoandrographolide to IL-6, glycyrrhetinic acid, oleanolic acid, ursolic acid, and wilforlide A to STAT3 are extremely high. In conclusion, the IL-6 and STAT3/IL-17signaling pathways could be critical regulative targets of HDW on LN.

## 1 Introduction

Systemic lupus erythematosus (SLE) is an autoimmune connective tissue disease with dysregulated autoimmune tolerance, production of multiple autoantibodies, and deposition of the immune complexes resulting in inflammation and multiorgan involvement (1). Among them, kidney involvement is the primary manifestation, and about 50%-70% of SLE patients develop lupus nephritis (LN) (2). Within ten years of an initial SLE diagnosis, 5-20% of LN patients develop end-stage kidney disease and the multiple comorbidities associated with immunosuppressive treatment, including infections, osteoporosis, and cardiovascular disease (3). LN is still a major cause of morbidity and death among patients with SLE (4).

A retrospective cohort study reported that combination therapy with Traditional Chinese medicine (TCM) might improve survival in SLE patients (5). Applying TCM can reduce GC use, the occurrence and severity of the side effects, and exert various pharmacological effects, such as reducing the deposition of immune complexes and reducing the production of inflammatory factors (6, 7). *Hedyotis diffusa* Willd (HDW) is a synonym of *Oldenlandia diffusa* (Willd.) Roxb, which is documented as a medicinal plant on http://www.theplantlist.org/. As a well-known traditional Chinese Medicine, it is also commonly used to treat LN by clearing away heat and detoxifying (8–10). Pharmacological studies show that HDW has antioxidative, anti-inflammatory, neuroprotective, and immunomodulating effects (11). The HDW extract had a protective effect against renal inflammation induced by LPS (12), but its specific mechanism in the treatment of LN remains unclear.

With the development of systems biology, network biology, and polypharmacology, the concept of network pharmacology was put forward by Andrew L Hopkins (13). The idea of TCM was consistent with those of network pharmacology. Many medical experts have successfully used network pharmacology to discover the practical components and the pharmacological mechanism of TCM herbs (14, 15). Up to 171 compounds have been isolated from HDW, including iridoids, anthraquinones, flavonoids, phenolics, volatile oils, and so on (11). Based on the known compounds in HDW, network pharmacology was applied in this study to screen out signaling pathways related to the treatment of LN.

Glucocorticoids (GC) are the first-line therapies for SLE. However, long-term GC application may result in unwanted effects that can be more harmful than the disease itself. Therefore, GC resistance and side effects remain a challenge in LN treatment (16). During the last five years, numerous reports have studied the inflammatory status of SLE patients, which is characterized by high production of IL-17 and resistance to GC suppression (17). GC even elevates IL-17 in certain diseases, such as asthma, Crohn’s disease, and SLE (18, 19). Signal transducer and activator of transcription 3 (STAT3), the critical transcription factor in Th17 cells, antagonizes glucocorticoid receptor functions (20). Moreover, GC enhances Th1/Th17 imbalance and STAT3 expression in SLE, which are also related to GC resistance (21). GC also elevates blood neutrophil counts and inhibits neutrophil apoptosis and neutrophils promoting Th17 cells (22, 23). Therefore, GC promotes the feedforward loop between STAT3/IL-17 activity and neutrophils.

The current study employs network pharmacological analysis to predict that HDW regulates the IL-17 signaling pathway. The MRL/lpr lupus model mice were used to evaluate HDW’s protective effect against renal damage in LN. Additionally, the therapeutic mechanism of HDW on LN was investigated. The article also explores the mechanism of HDW to mitigate the adverse effects and improve the therapeutic effects of GC treatment. Furthermore, the critical compounds in HDW were identified with literature mining and molecular docking methods.

## 2 Materials and methods

### 2.1 Materials and equipment

The whole herb of HDW (191202) was obtained from the Zhejiang Chinese Medical University Traditional Chinese Medice Co. Ltd. (Hangzhou, China). The Cytometric Bead Array (CBA) Mouse Inflammation Kit (552364) was purchased from BD (New Jersey, USA). HRP-labelled goat anti-mouse IgG (H+L) antibody was purchased from ImmunoWay (Texas, USA). The specific antibodies against STAT3 (sc-8019) and IL-17 (sc-374218) were purchased from Santa Cruz Biotechnology (Texas, USA). The MPO Polyclonal Antibody (AP-73534) was purchased from Abcepta (Shanghai, China), and Ly-6G/Ly-6C Monoclonal Antibody (14–5931–85) was purchased from Santa Cruz Biotechnology (Birmingham, UK). The goat anti-rabbit (C10324-01) and rabbit anti-mouse (I20012C) secondary antibodies were purchased from LICOR (Nebraska, USA) and Tuling (Hangzhou, China), respectively.

### 2.2 Preparation of HDW extract

When *Hedyotis diffusa* is used to treat SLE patients clinically, it is almost taken as a decoction. The “Jieduquyuzishen” prescription we use in the clinical treatment of SLE patients also includes an aqueous extract of *Hedyotis diffusa* (24). The whole herb of HDW was soaked in cold water for 30 minutes, then boiled for 30 minutes. After filtrating with 3M filtration paper, the HDW extract was concentrated to 1 g/mL (1 g crude drug/mL) with a rotary evaporator (R2O2). This aqueous extract of HDW was used in this study.

### 2.3 UPLC-Q-TOF-MS analysis of HDW

A total of 3 μL HDW extract (4 mg/mL) was loaded into an ACQUITY UPLC HSS C18 column (100×2.1 mm, 1.7μm) of an Ultra-High-Performance Liquid Chromatography System. The system was operated at a flow rate of 0.3 mL/min. The temperatures of the column and autosampler were maintained at 40°C and 8°C, respectively. The eluate-mobile phase comprised of 0.1% formic acid water solution (solution A) and acetonitrile (solution B) in a gradient eluate mode as follows: 0–2 min, 95% B; 2–17 min, 95% B; 17–20 min, 45% B; 20-21 min, 5% B; 21–24 min, 5% B; and 13.1–15 min, 5% B. Time-of-flight mass spectrometry was performed with TurboIonSpray as ionization source, and positive and negative ion scanning modes were applied for analysis. Finally, the results were comparatively analyzed with SCIEX OS software.

### 2.4 Network pharmacology analysis

#### 2.4.1 Collection of candidate ingredients and potential targets

The ingredients in HDW were screened using the Traditional Chinese Medicine Systems Pharmacology Database and Analysis Platform (TCMSP) (https://tcmspw.com/tcmsp.php). This website provides information on the ingredients, such as chemical structure and the parameters of absorption, distribution, metabolism, and excretion (ADME). The active compounds with oral bioavailability (OB) ≥ 30% and drug-like (DL) ≥ 0.18 were selected for further studies. We compared the components screened by UPLC-Q-TOF-MS analysis with the components obtained from the TCMSP database, after taking the intersection, the final 15 components were included for network pharmacology analysis. The information on the selected ingredients is listed in **Supplemental Table 1**.

In the present study, the TCMSP database, PharmMapper server (based on the reverse pharmacophore matching principle), SEA online search tool (based on the principle of target ligand structure similarity), and STITCH database (based on the principle of compound and protein interaction) were used to explore the potential targets of the selected ingredients in HDW. Subsequently, the targets screened by the four databases were integrated and the target information, such as gene code and name, was standardized and de-duplicated with the UniProt database (https://www.uniprot.org/). Finally, the active ingredients-targets network was constructed with Cytoscape 3.9.0 software.

#### 2.4.2 Collection of disease targets for LN

The specific LN targets were collected from the GEO and the current online disease databases. The microarray data GSE113342 and GSE104948 were downloaded from the Gene Expression Omnibus database (GEO, http://www.ncbi.nlm.nih.gov/geo/). These databases consist of 19 standard samples, 28 renal tubule samples, and 60 glomerular samples from LN patients. The Bioconductor/R limma package screened differentially expressed genes (DEGs) between control and patients. Furthermore, the LN targets were obtained from five currently available databases using “Lupus Nephritis” as the keyword: DrugBank (https://go.drugbank.com/), OMIM (https://www.omim.org/), GAD (https://geneticassociationdb.nih.gov/), TTD (http://db.idrblab.net/ttd/), GooLGeN (http://ci.smu.edu.cn/genclip3/analysis.php). Finally, the collected targets were integrated to construct a disease-target network with Cytoscape 3.9.0 software.

#### 2.4.3 Protein-protein interaction (PPI) network construction

The data for PPI network construction were exported from six currently available PPI databases (25), including The Biological General Repository for Interaction Datasets (BioGRID), the Molecular INTeraction Database (MINT), the Biomolecular Interaction Network Database (BIND), the Database of Interacting Proteins (DIP) and the Human Protein Database (HPRD), searched by BisoGenet, a Cytoscape plugin. Firstly, an interactive network for the putative HDW drug targets and known LN-related targets was constructed to determine their interactions (26). To screen the key targets, the plug-in CytoNCA was applied to analyze the topological properties of each node (HDW-LN target) in the interaction network by calculating six metrics “betweenness centrality (BC),” “degree centrality (DC),” “eigenvector centrality (EC),” “closeness centrality (CC),” “network centrality (NC),” and “local average connectivity (LAC)”. The definition and calculation formula of these six parameters represent the topological crucial of the nodes. Afterward, the PPI network of ingredients in HDW and LN was visualized using Cytoscape software (Version 3.9.0). The PPI networks of the targets of HDW only was directly constructed with the STRING database. The results were then imported into Cytoscape 3.9.0 software to identify key genes by screening with the Degree algorithm of the CytoHubba plugin and visualizing the interactions among the proteins.

#### 2.4.4 KEGG pathway analysis and molecular docking

The Kyoto Encyclopedia of Genes and Genomes (KEGG) pathway enrichment analysis of the ClueGO plugin in Cytoscape 3.9.0 software was employed to analyze the function of the targets and the involved metabolic pathways. The Homo species, the ontology reference, and a Kappa score of 0.35 were selected in this analysis.

The crystal structures of the target proteins were downloaded from the PDB database (RCSB PDB, https://www.rcsb.org). Afterward, the pro-ligand and target proteins were separated using pymol software and subjected to dehydration and hydrogenation. Then the corresponding ingredients were docked separately with these targets by Autodock 1.5.7 software. Finally, the conformation with the lowest binding energy was visualized by PyMOL.

### 2.5 Animals and treatment

Thirty MRL/lpr lupus mice were randomly divided into five groups: model group (M), Prednisone treated group (PAT), HDW low-dose group (HDW-L), HDW middle-dose group (HDW-M), HDW high-dose group (HDW-H), with six mice in each group. Six C57BL/6 mice were assigned to the control group (CK). The mice in each group were given ordinary feed. The control and model groups were intragastrically administrated (i.g.) the identity volume saline. The PAT group was given 6 mg prednisone per 1kg bodyweight every day. The HDW dose of the HDW-L, HDW-M, and HDW-H groups was i.g. 2.5 g, 5 g, and 10 g per 1kg body weight every day, respectively. This experiment lasted 40 days. The mouse urine was collected five times over the last week, and the urine samples were mixed with sodium azide and then stored in a -80 °C refrigerator for protein concentration detection.

Each group of mice was fasted and weighed at the end of the experiment. Mice were anesthetized with tribromoethanol (Avertin) with a 1.25% avertin solution dose of 0.02 mL per gram body weight. The peripheral blood was collected in an EDTA-K2 anticoagulant tube. The white blood cells, including Lymphocytes (LYM) and neutrophils (NEUT), were analyzed by The ProCyte Dx analyzer at the Animal Experimentation Center of Zhejiang Chinese Medical University. After staining at room temperature for 60 minutes, the serum was obtained by centrifuging at 3000 rpm, 4°C for 10 minutes. The CBA Mouse Inflammation Kit performed cytokine assays on mouse serum and the anti-nuclear antibodies in serum were tested by following the ELISA kit instructions. Subsequently, the kidneys were collected and fixed in a neutral formaldehyde solution, dehydrated by gradient ethanol, and sliced (4 μm) after embedding in paraffin. Finally, hematoxylin and eosin staining (H&E staining) and periodic acid-Schiff staining were performed in sequence (PAS staining) for histopathological observation.

### 2.6 Immunohistochemical and immunofluorescence staining

For immunohistochemical analysis, the sliced paraffin-embedded kidney sections were dewaxed and rehydrated. The endogenous peroxidase was inactivated before being blocked with a goat serum solution for 30 minutes. The STAT3 and MPO were hybridized with their specific antibodies overnight at 4°C and detected with secondary antibody and diluted Sav-HRP conjugates to develop the visible color signal. After washing with PBS three times, these sliced sections were stained with DAB to reveal the color. Then these sections were thoroughly washed in hematoxylin for 5 min and photographed by the microscope. For immunofluorescence staining analysis of IL-17 or Ly6G, the sliced sections were first hybridized with specific antibodies and detected with fluorescein-labeled goat anti-mouse secondary antibodies under darkness. After rinsing with PBS buffer, the antifade mounting medium was added. Finally, these slices were observed and photographed with a fluorescence microscope.

### 2.7 Preparation of the bone marrow neutrophil and NETosis formation detection

The bone marrow cells were prepared according to the kit specification. Briefly, the reagents and cell suspension were added sequentially in a 15 mL centrifuge tube with a gradient interface. The tube was centrifuged at 400-550 g for 25 minutes. Then the neutrophil layer was transferred into another 15 mL centrifuge tube and the cells were cleaned with a washing solution. The cells were collected by centrifugation and resuspended with the specific medium for NETosis protective experiments. NETosis formation was observed under the microscope after DAPI staining.

### 2.8 Statistical analysis

All data were presented as mean ± standard deviation. T-tests for the independent sample data were used for pairwise comparisons with SPSS Statistics 19.0 software. A *P-value* of <0.05 was considered statistically significant.

## 3 Results

### 3.1 Network pharmacology analysis of HDW on the treatment of LN

Due to the multi-components and multi-target synergistic effects of traditional Chinese medicine, network pharmacology is used to guide the research on the specific mechanism of action of TCM (27).

**Figure 1:**
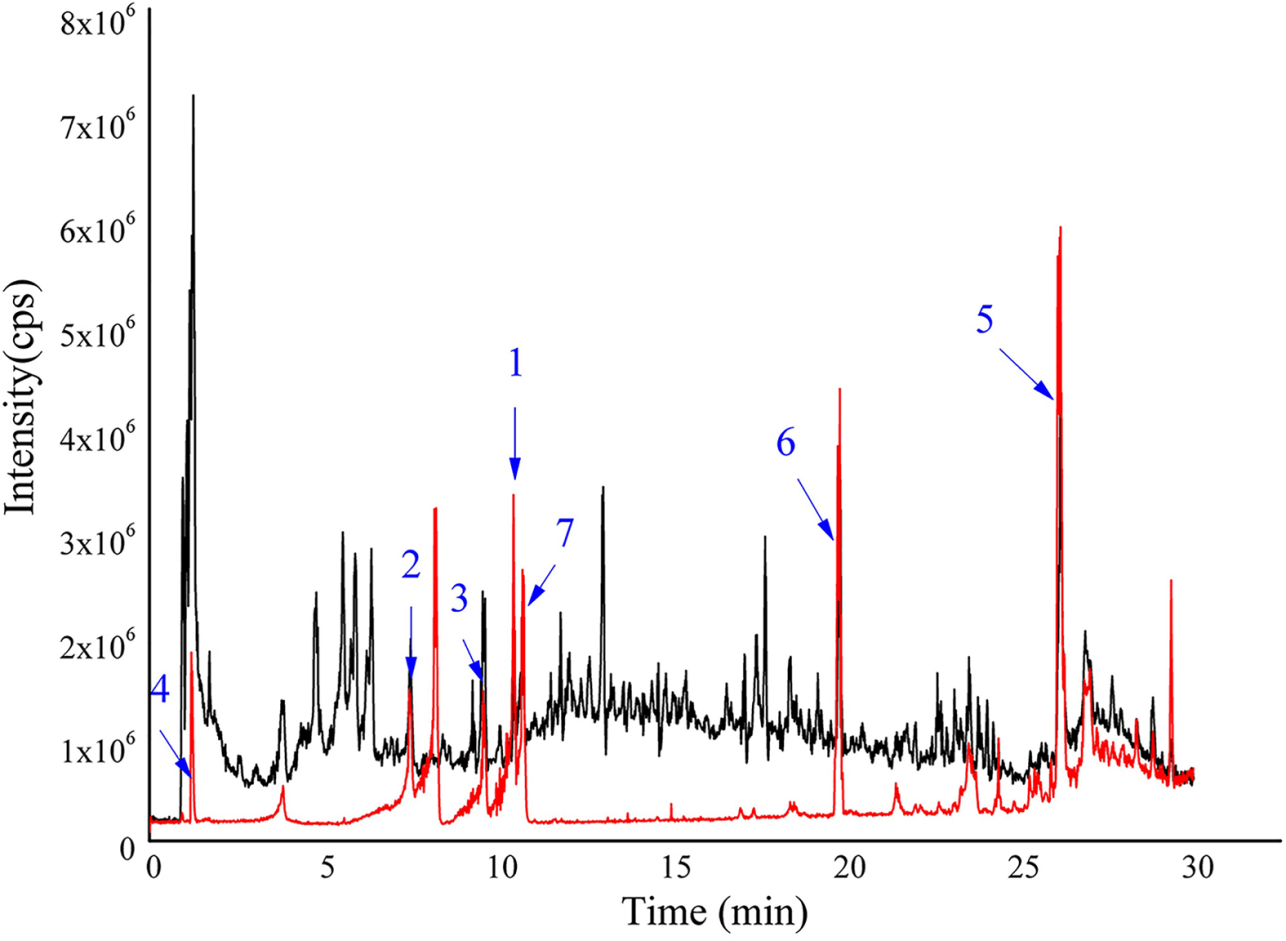
Total ion chromatograms of HDW and seven standards were obtained through UPLC-Q-TOF-MS/MS. HDW sample shown with black; mixed standards marked with red, 1-7 are scopoletin, asperuloside, p-coumaric acid, deacetylasperulosidic acid, ursolic Acid, 2-hydroxy-3-methylanthraquinone and rutin, respectively.

**Figure 2:**
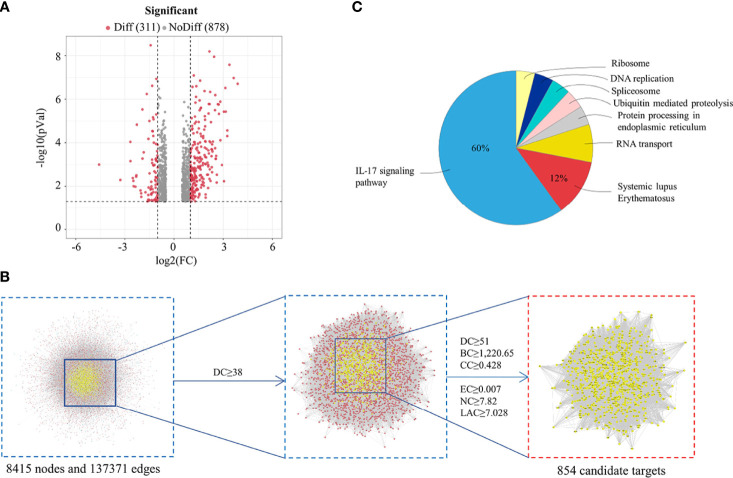
Analysis of HDW treatment LN Based on Network Pharmacology. **(A)** Differential expression volcano map shows the differentially expressed genes in LN samples; grey pixels represent genes where the difference in expression is not significantly different, red pixels represent significant ones; the X-axis displays log2 fold change, and the Y-axis indicates the -log10 *p-value*. **(B)** Topological analysis of the protein-protein interaction network. **(C)** KEGG enrichment analysis of HDW targets in treating LN.

Firstly, a UPLC-Q-TOF-MS/MS analysis of HDW was performed, identifying 49 distinct components in NEG mode, including seven characteristic compounds reported in the literature (**Figure 1**; **Supplemental Figures 1A–G**). Finally, 15 typical active ingredients of HDW were selected for network pharmacology analysis (**Supplemental Table 1**). Then the TCMSP, PharmMapper server, SEA online search tool, and STITCH database were used to mine the potential targets of HDW active ingredients, obtaining 2674 target genes. The active compounds (compound ID) and potential targets were imported into Cytoscape software to construct a compound-targets network diagram (**Supplemental Figure 2**). Next, to further explore the targets of the active compounds of HDW in SLE, we collected information about the disease targets of LN. The chips GSE113342 and GSE104948 in the GEO database consist of 19 standard samples, 28 renal tubule samples and 60 glomerular samples from patients with LN. A total of 560 DEGs were screened through the Bioconductor/R limma package. (**Figure 2A**; **Supplemental Figure 3**). Furthermore, this study also collected the disease target data of LN from 5 online databases, DrugBank, OMIM, GAD, TTD, and GooLGeN, yielding a total of 782 targets.

To further clarify the role of HDW in the prevention and treatment of LN, the Merge function of Cytoscape software was used to construct the compounds-targets-Disease network PPI by the targets of the active compounds and the disease targets information; the PPI network consisted of 1505 nodes and 65589 edges. Next, CytoNCA, a plug-in of Cytoscape software, was used to calculate the topological characteristics of each hub (such as “BC,” DC,” “EC,” “CC,” “NC,” and “LAC”). In total, 854 core targets were screened out (**Figure 2B**), and the ClueGO plug-in was used to perform KEGG pathway enrichment analysis. The ingredients KEGG enrichment analysis revealed that HDW primarily regulates the IL-17 pathway in LN treatment (**Figure 2C**).

### 3.2 HDW attenuated inflammatory cytokines and autoantibodies in the serum of MRL/lpr mice

**Figure 3:**
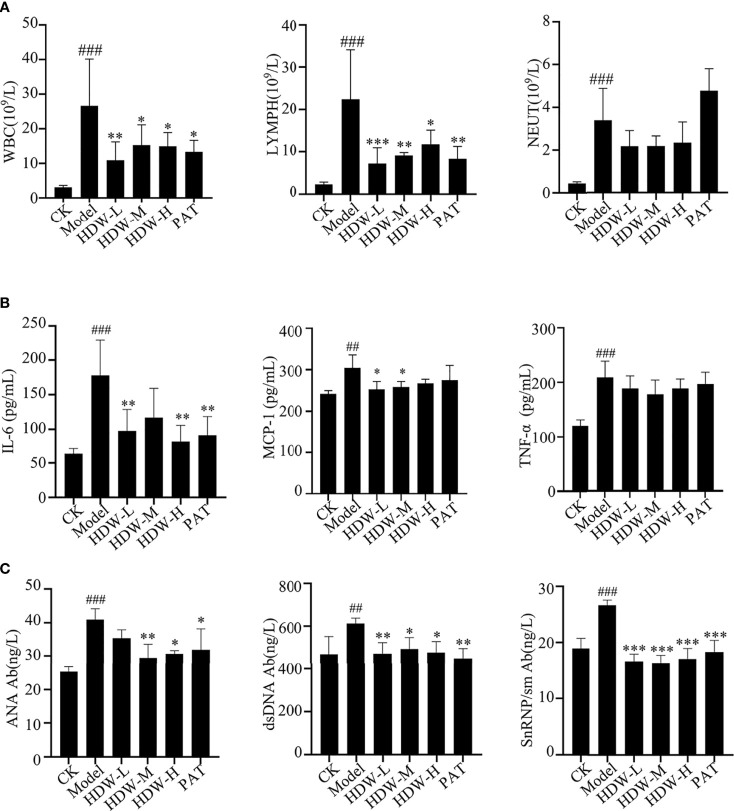
Effect of HDW on hematologic-related indices in MRL/lpr mice. **(A–C)** The effect of HDW on white blood cell parameters **(A)**, Serum Cytokine Profiles **(B)**, and the autoantibodies **(C)** of MRL/lpr mice. Six mice per group were included in the statistics. #Indicates the difference between the model group and the control group, ^##^ *P* < 0.01, ^###^ *P* < 0.001; * Indicates the difference between the treatment group and the model group *P < 0.05, **P < 0.01, ***P < 0.001.

MRL/lpr mouse is a model mouse with spontaneous lupus, in which mutations in the Fas gene lead to massive lymphoproliferation (lpr) and accelerate autoimmunity. Some pathological responses are similar to those of SLE patients, such as renal damage and abnormal cytokine expression etc (28). The pathogenesis of SLE involves multiple systemic injuries and a variety of autoantibodies, which can damage the peripheral blood circulation system to varying degrees and cause hematological system damage. The blood cells of MRL/lpr mice were first detected, revealing that the leukocyte and lymphocyte levels were significantly reduced in the HDW and PAT groups compared with the M group. Interestingly, the level of neutrophils was reduced in HDW mice but not in the PAT group (**Figure 3A**).

Cytokines play an essential role in the autoimmune response. They are produced by T cells, B cells, and macrophages (29). Here, the cytokine levels in the serum of different groups of mice were detected. The results showed that the contents of IL-6, MCP-1, and TNF-α in the model group were significantly increased compared to C57BL/6 mice. Compared with the model group, the three HDW and PAT groups showed remarkably lower IL-6 and MCP-1 levels. Moreover, the concentration of TNF-α was also depressed in these groups, but the difference was not statistically significant. (**Figure 3B**).

Serum anti-nuclear antibody levels are used clinically as one of the critical criteria for determining the SLE disease activity score. This study detected the anti-nuclear antibodies in mice serum with an ELISA kit. The levels of anti-ANA, anti-dsDNA, and anti-snRNP/Sm antibodies were significantly higher in the serum of mice in the M group compared with the CK group (*P* < 0.005). These anti-nuclear antibodies were substantially lower in the HDW groups and PAT group compared with the M group (**Figure 3C**).

### 3.3 HDW inhibits the expression of STAT3/IL-17 in the kidney to prevent neutrophil infiltration

**Figure 4:**
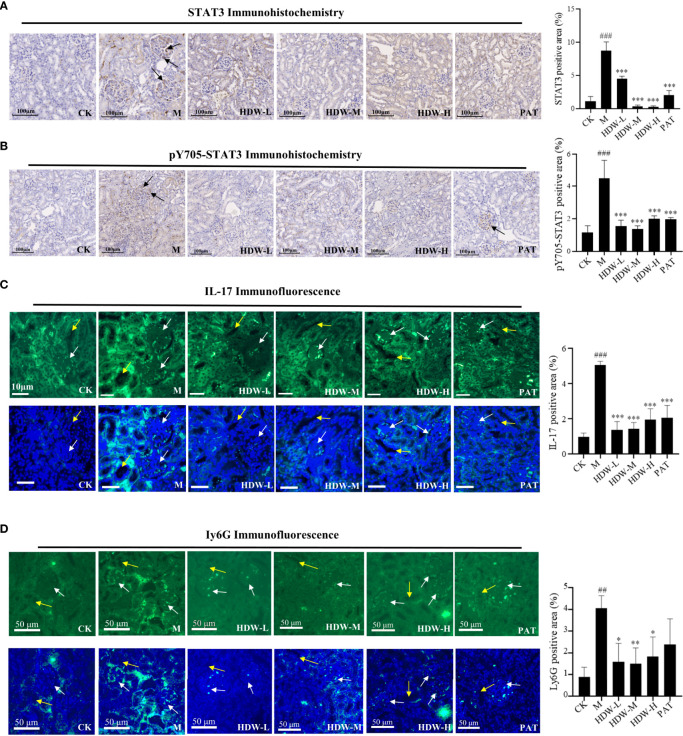
Effect of HDW on the expression of STAT3, IL-17, and Ly6G in the kidney of MRL/lpr mice. **(A)** STAT3 immunohistochemistry staining. **(B)** pY705-STAT3 immunohistochemistry staining. Representative immunohistochemistry of STAT3 or pY705-STAT3 staining exhibited brown staining. The brown signals indicated by the black arrow are in the glomerular region. **(C)** Immunofluorescent staining of IL-17 **(D)** Immunofluorescent staining of Ly6G. White arrows indicate the glomerular region, and the yellow arrows indicate the tubular region. The right histogram shows the relative expression of STAT3, pY705-STAT3, IL-17, and Ly6G respectively. The expressions were quantified by pixel counting in three regions of each section. #Indicates the difference between the model group and the control group ^##^ *P* < 0.01, ^###^ *P* < 0.001; * Indicates the difference between the treatment group and the model group *P < 0.05, **P < 0.01, ***P < 0.001.

The network pharmacology results predicted that the mechanism of HDW intervention in lupus nephritis might involve the IL-17-related pathway. Upregulated expression of STAT3/IL-17 is observed in patients with SLE, and a positive correlation was found between IL-17 expression and STAT3. IL-17 expression is significantly correlated with STAT3 phosphorylation, and IL-17 can activate STAT3 to promote cell growth and inflammation (30, 31). In addition, IL-6 is an immunomodulatory cytokine involved in the inflammatory response and the proliferation and differentiation of Th17 cells (32). The present study demonstrated that HDW could significantly inhibit IL-6 expression in MRL/lpr mice, suggesting that the IL-6/STAT3 pathway might be involved. To investigate whether STAT3 influences IL-17 production in MRL/lpr mice, STAT3, pY705-STAT3, and IL-17 *in situ* immunofluorescence staining was performed on the kidneys of MRL/lpr mice. The results showed that STAT3, pY705-STAT3, and IL-17 antibody deposition were significantly increased in the M group (**Figures 4A** **–C**). Compared with the M group, inhibited STAT3 antibody deposition and the immunofluorescence expression of IL-17 in the three HDW groups and the PAT group were observed.

Activation of IL-17 stimulates many inflammatory genes, including pro-inflammatory cytokines and neutrophil-specific chemokines (33). Furthermore, IL-17 promotes the recruitment of other immune cells by upregulating the expression of CCL2, MCP-1, and CCL7. The interactions between immune cells and organ tissue cells exacerbate inflammation and induce a specific pro-inflammatory cytokine environment (34). Subsequently, immunofluorescence staining of Ly6G, the surface antigen of neutrophils, was performed to explore the effects of HDW on neutrophil infiltration by inhibiting IL-17 production in kidneys. Compared with the CK group, Ly6G was significantly increased in the model group, while decreased Ly6G expression was observed in the HDW and PAT groups (**Figure 4D**). From the position of the fluorescent signal in **Figure 4D**, Ly6G positive signal area is concentrated in the periphery of the glomerulus, and the position of the renal tubule. It indicates that HDW may interfere with the chemotaxis of neutrophils in the kidney, ultimately affecting the production of IL-17.

### 3.4 HDW reduces the pathological damage of kidneys in MRL/lpr mice by inhibiting NETosis formation

**Figure 5:**
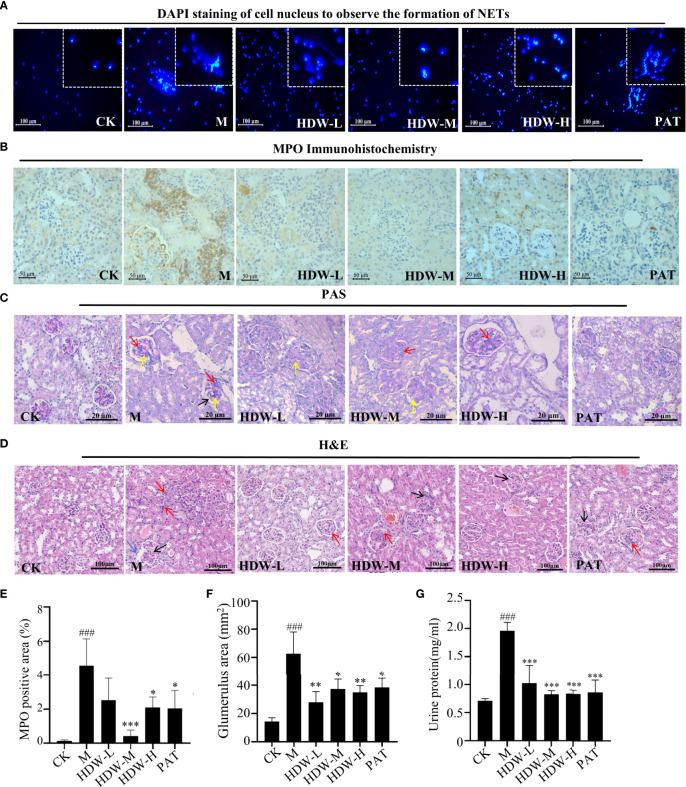
The effect of HDW on NETosis formation and histopathological changes in MRL/lpr mouse kidneys. **(A)** The Effect of HDW on NETosis formation of bone marrow neutrophils. DAPI staining is shown in blue. **(B)** MPO immunohistochemistry staining. Brown deposits are protein-positive deposits. **(C)** Representative periodic acid-Schiff (PAS)-stained sections: red arrows mark increased inflammatory cells; yellow arrows mark thylakoid hyperplasia; black arrows mark basement membrane thickening. **(D)** Representative hematoxylin and eosin (H&E) sections: red arrows mark increased inflammatory cells; black arrows mark thylakoid hyperplasia; blue arrows mark glomerular crescent. **(E)** The MPO staining was quantified by pixel counting of sections using ImageJ. **(F)** Areas of glomerular volume. **(G)** Urinary protein levels in MRL/lpr mice. #Indicates the difference between the model group and the control group, ^###^ *P* < 0.001; * Indicates the difference between the treatment group and the model group *P < 0.05, **P < 0.01, ***P < 0.001.

The present study demonstrated that HDW reduced the expression of Ly6G in the kidneys of MRL/lpr mice. Excess neutrophil extracellular traps (NETosis) damage normal tissues and induce inflammation and immune injury to organs, such as vessels, kidneys, etc., if it is not treated in time (35). MRL/lpr mouse bone marrow neutrophils were isolated and cultured *in vitro* to validate the inhibitory activity of HDW on NETosis formation. The results demonstrated that even low-dose HDW (2.5g/kg•d) still showed significant inhibitory properties. In the PAT group, no significant inhibition of NETosis formation was observed. This result suggests that *Hedyotis diffusa* has a different mechanism in the treatment of SLE compared with glucocorticoids (**Figure 5A**). NETosis structures are composed of a network of chromatin strands associated with various neutrophil-derived proteins, including the enzyme myeloperoxidase (MPO). Immunohistochemical staining of MPO showed heavy deposition of MPO antibodies in the model group, while MPO expression was significantly reduced in the HDW and PAT groups (**Figures 5B** **, E**). This suggests that HDW may reduce the inflammatory response in the kidney by reducing neutrophil infiltration and NETosis formation.

Moreover, a histopathological examination of the kidneys of MRL/lpr mice was carried out. Compared with the CK group, the glomerulus of MRL/lpr mice was intumescent, with apparent inflammatory cell infiltration. A large amount of inflammatory cell infiltration was observed in the kidney interstitium, with the proliferation of glomerular mesangial cells and thickened basement membrane. The mice in the HDW and PAT groups showed attenuated pathological changes compared with the model group **(** **Figures 5C, D, F** **)**.

Urine protein testing and kidney biopsy were used to determine renal damage and the severity of renal impairment (36). Compared with normal C57 mice, the urine protein content was significantly increased in the model group. Compared with the model group, significantly reduced urine protein content was observed in the PAT group, the middle and high dose groups of HDW (**Figure 5G**).

### 3.5 Network pharmacological analysis of active ingredients in HDW on the regulation of the IL-6/STAT3 pathway

**Figure 6:**
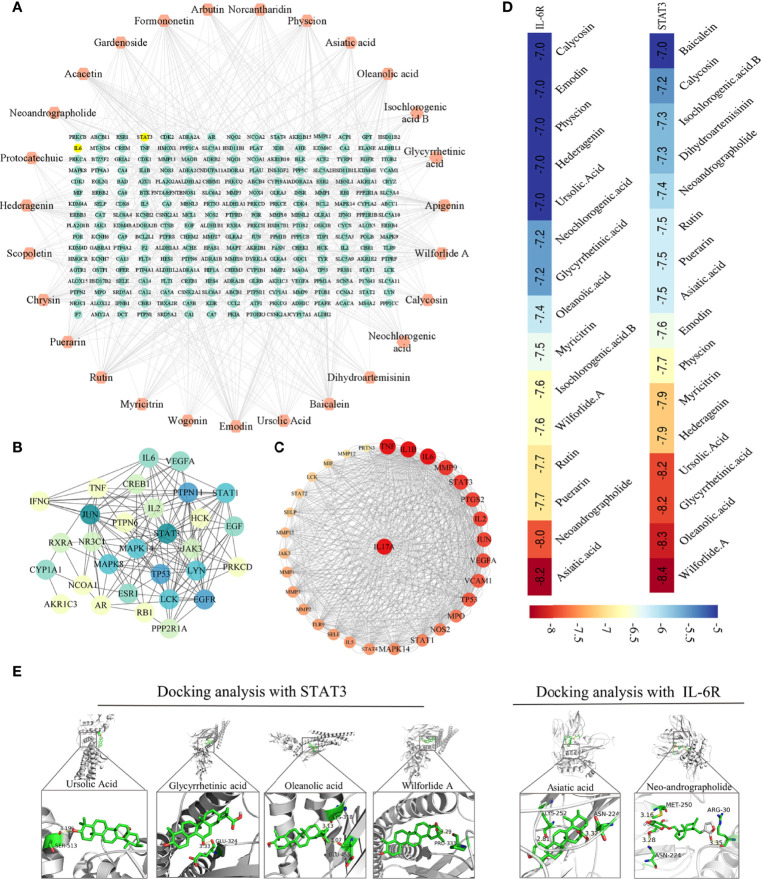
Network pharmacological analysis of active ingredients in HDW on the regulation of the IL-6/STAT3 pathway. **(A)** The 27 compounds-targets Network Diagram. **(B)** PPI of the top 30 targets filtered by degree algorithm. **(C)** PPI Map of the top 30 targets and IL-17, the targets are sorted in descending order in a circular layout based on the number of degrees. The size of the target reflects the number of degrees. **(D)** The compounds whose binding energy with STAT3 or IL-6R was lower than -7.0, with colors closer to red indicating a lower score. **(E)** The docking pose of STAT3 with glycyrrhetinic acid, oleanolic acid, ursolic acid, and wilforlide A (left); The docking pose of IL-6R with asiatic acid and neoandrographolide (right).

The above study showed that the interventional effect of HDW on LN may involve the STAT3/IL-17 pathway. Literature mining on the active compounds of HDW was carried out, revealing that 27 compounds regulate the IL-6/STAT3 signaling pathway. The regulatory targets of these 27 compounds were further constructed through network pharmacology (**Figure 6A**). Next, the compound targets were analyzed by degree algorithm with the cytohubba plug-in, and the top 30 targets were screened for visualization. Most of these gene targets are closely related to SLE disease (**Figure 6B**). The degree of association of these top 30 gene targets with IL-17 analysised by PPI. The top five associated genes are TNF, IL1B, IL-6, MMP9, and STAT3 (**Figure 6C**). Animal experiments have shown that HDW significantly inhibits the IL-6/STAT3 pathway. Both IL-6 and STAT3 have recently emerged as central regulators of the differentiation and function of Th17 cells *via* a positive feedback loop enhancing the expression and activation of IL-6. Therefore, these 27 compounds were molecularly docked with IL-6R and STAT3 proteins, respectively; it is generally considered that lower binding energy results in higher affinity and binding property (**Figure 6D**). The molecular docking results showed that asiatic acid and neoandrographolide bind to the IL-6R protein with less than -8kcal/mol. The other four compounds, glycyrrhetinic acid, oleanolic acid, ursolic acid, and wilforlide A, bind to the STAT3 protein with less than -8kcal/mol (**Figure 6E**).

## 4 Discussion

LN is one of the most severe complications of SLE, and it is also the main factor determining the long-term prognosis and death of SLE patients (37). There is no independent disease name for LN in Chinese medicine. It is believed that LN belongs to the categories of “Kidney Bi” and “Red Butterfly” (38). HDW is a traditional Chinese medicine for clearing away “heat” and detoxifying, commonly used to treat LN (39). There have been many relevant studies investigating the properties of HDW, such as antibacterial, anti-inflammatory, anti-tumor, and improving immunity (40, 41). UPLC-Q-TOF-MS was used to identify the chemical components of HDW, and the network pharmacology analysis showed that HDW treated LN by regulating the IL-17 pathway. Recent studies in human SLE and animal models indicate the crucial role of IL-17 in LN pathogenesis. A high level of IL-17 correlated with poor outcomes after immunosuppressive therapy in patients with LN (42). The mechanism of HDW may involve the regulation of the IL-17 signaling pathway, exhibiting synergistic effects for the clinical treatment of LN.

An important finding in this study is that HDW administration effectively reduced the content of serum IL-6 in MRL/lpr mice. IL-6 production and dysregulation are associated with chronic inflammatory diseases and autoimmunity (43). IL-6 triggers the dimerization of the IL-6 receptor and leads to the activation of the STAT3. STAT3 induces Th17 cell differentiation and further enhances IL-17 and IL-6 expression through a positive feedback loop (44). Urine IL-17 levels are increased in SLE patients and are associated with LN activity (45, 46). Here, HDW significantly reduced STAT3 and IL-17 expression in renal tissue, consistent with HDW’s decreased serum IL-6 secretion. IL-17 induces the production of additional inflammatory cytokines to promote the recruitment of neutrophils to the inflamed organs (47). MCP-1 is a small molecule cytokine of the C-C chemokine family, which induces monocytes and endothelial cells to express adhesion molecules, causing various inflammatory cells to accumulate at the lesion site. It plays a vital role in LN kidney injury (48). In addition to reducing the expression of IL-17 in the kidney, HDW reduced MCP-1 levels in the serum of MRL/lpr mice. Consistent with the decreased IL-17 and MCP-1 levels, HDW reduced inflammatory cell infiltration in renal tissue, which is a major indicator of improvement in pathological renal damage.

**Figure 7:**
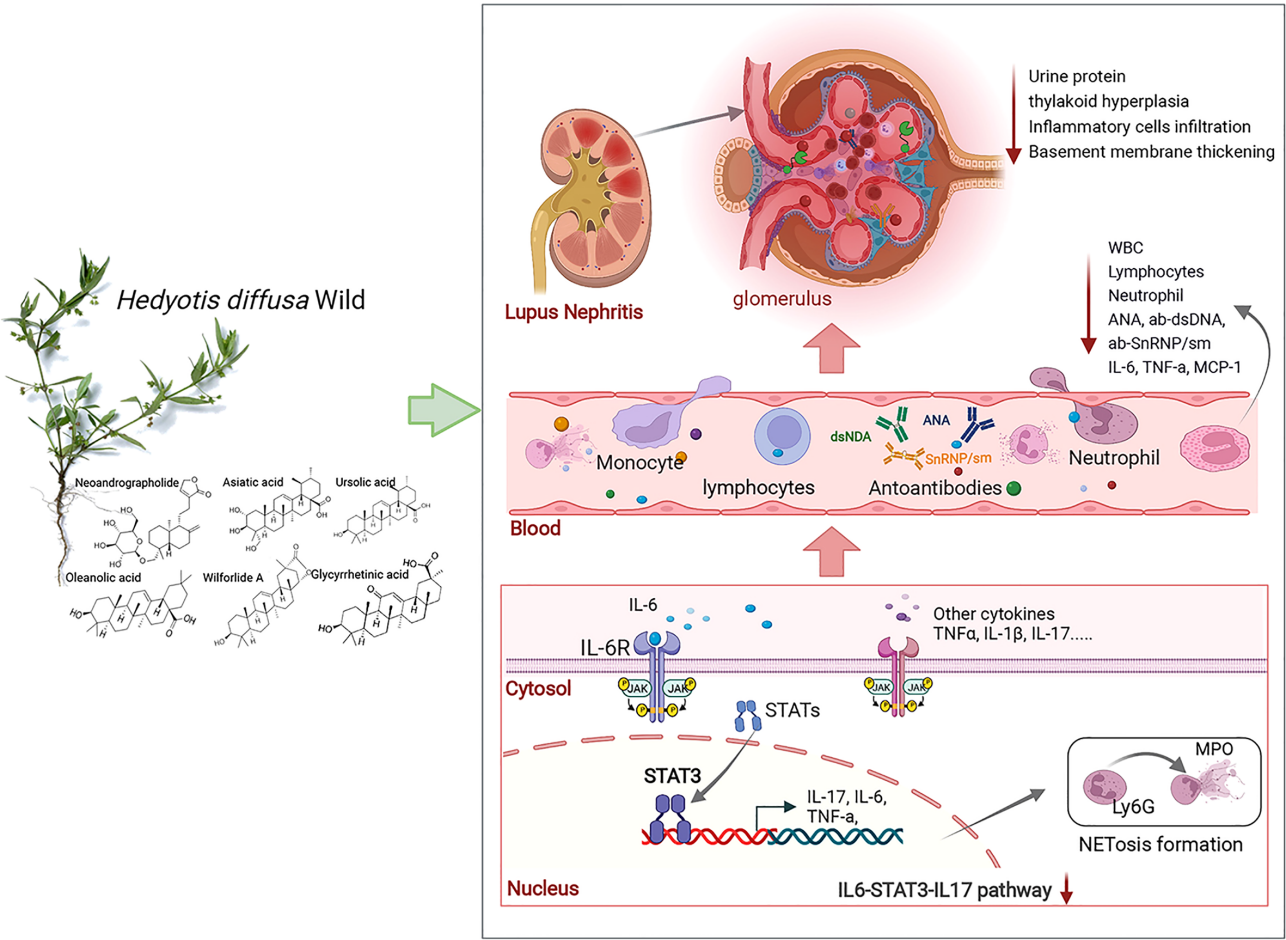
HDW exerts a protective effect against LN *via* STAT3/IL-17. IL-6, interleukin-6; MCP-1, macrophage chemoattractant protein-1; IL-17, interleukin-17; Ly6G, lymphocyte antigen 6complex, locus g; MPO, Myeloperoxidase.

The imbalance between the formation and clearance of NETosis and the excess NETosis deposited in tissues and organs can amplify the inflammatory response and promote autoimmune disease progression (49). The level of NETosis in SLE patients was positively correlated with the SLE disease activity index (SLEDAI). IL-17 induces NETosis formation through direct action on neutrophils. It has been reported that IL-17 causes NETosis in animals prone to lupus (31, 50). In this study, bone marrow neutrophils were extracted and cultured *in vitro*; the results confirmed that HDW inhibited the formation of NETosis. The NETosis induction was found to be dependent on MPO activity and deposited MPO in glomeruli is elevated in ANCA-associated glomerulonephritis (35). HDW inhibited MPO deposition in renal tissue, indicating its therapeutic effect on LN. The pathological analysis further supported that HDW reduced urinary protein production and renal injury. This may be one of the essential mechanisms of HDW in treating LN (**Figure 7**). Furthermore, persistent proteinuria in SLE patients contributes to an increased risk of progressive chronic kidney disease (51). We conclude that HDW reduced neutrophil infiltration and NETosis formation by regulating the STAT3/IL-17 pathway. Many studies suggested that tumor-induced NETosis may be a promoter of cancer-associated pathology (52, 53). As we know, *Hedyotis diffusa* Willd. is also one of the most renowned herbs for cancer treatment in traditional Chinese medicine (54). The inhibition of NETosis formation by *Hedyotis diffusa* may also be an important mechanism for its curative effect on various cancers.

Finally, the ingredients in the aqueous extract of *Hedyotis diffusa* that regulate STAT3, IL-17, and IL-6 have been identified through literature mining and network pharmacology. Six chemical components, asiatic acid, neoandrographolide, glycyrrhetinic acid, oleanolic acid, ursolic acid, and wilforlide A in HDW, may be effective components for the prevention and treatment of LN. Asiatic acid is the main active ingredient of *Centella asiatica*. Interestingly, *Centella asiatica* and *Hedyotis diffusa* Willd are the two herbs for clearing away heat and detoxification in the Jieduquyuzishen Prescription, which is used for the clinical treatment of SLE (55). Network Pharmacology combining animal experiments provide a visual evaluation method for the complex components of Chinese herbal medicine and the mechanism of disease treatment. The results of our study provided mechanism support for the clinical application of HDW on LN.

## 5 Conclusion

Our results evaluated the activity of *Hedyotis diffusa* Willd in improving the blood biochemical parameters and pathological kidney changes in lupus-like mice. STAT3/IL-17 may be a potential pathway target of *Hedyotis diffusa* Willd to treat lupus nephritis.

## Data Availability Statement

The original contributions presented in the study are included in the article/**supplementary material**. Further inquiries can be directed to the corresponding author.

## Ethics Statement

The animal study was reviewed and approved by Laboratory Animal Management and Welfare Ethical Review Committee of Zhejiang Chinese Medical University.

## Author Contributions

YL and TD contributed equally to this paper. YL and TD performed the experiments and designed figures and tables. JC and JJ designed figures and tables. BD, WW, and WG revised the manuscript, and YF and LX supervised the research and revised the first draft of the manuscript. All authors contributed to the article and approved the submitted version.

## Funding

This research was supported by the National Natural Science Foundation of China (NO.81673857) and the Natural Science Foundation of Zhejiang Province (NO.LY22H100003).

## Conflict of Interest

The authors declare that the research was conducted in the absence of any commercial or financial relationships that could be construed as a potential conflict of interest.

## Publisher’s Note

All claims expressed in this article are solely those of the authors and do not necessarily represent those of their affiliated organizations, or those of the publisher, the editors and the reviewers. Any product that may be evaluated in this article, or claim that may be made by its manufacturer, is not guaranteed or endorsed by the publisher.
