# Supplementary material for: The protective capability of Hedyotis diffusa Willd on lupus nephritis by attenuating the IL-17 expression in MRL/lpr mice
Source: Front Immunol. 2022 Jul 25;13:943827. doi: 10.3389/fimmu.2022.943827 (PMC9359319; doi:10.3389/fimmu.2022.943827)
Supplement: Supplementary file 1 [file DataSheet_1.docx]

***Supplementary Material***
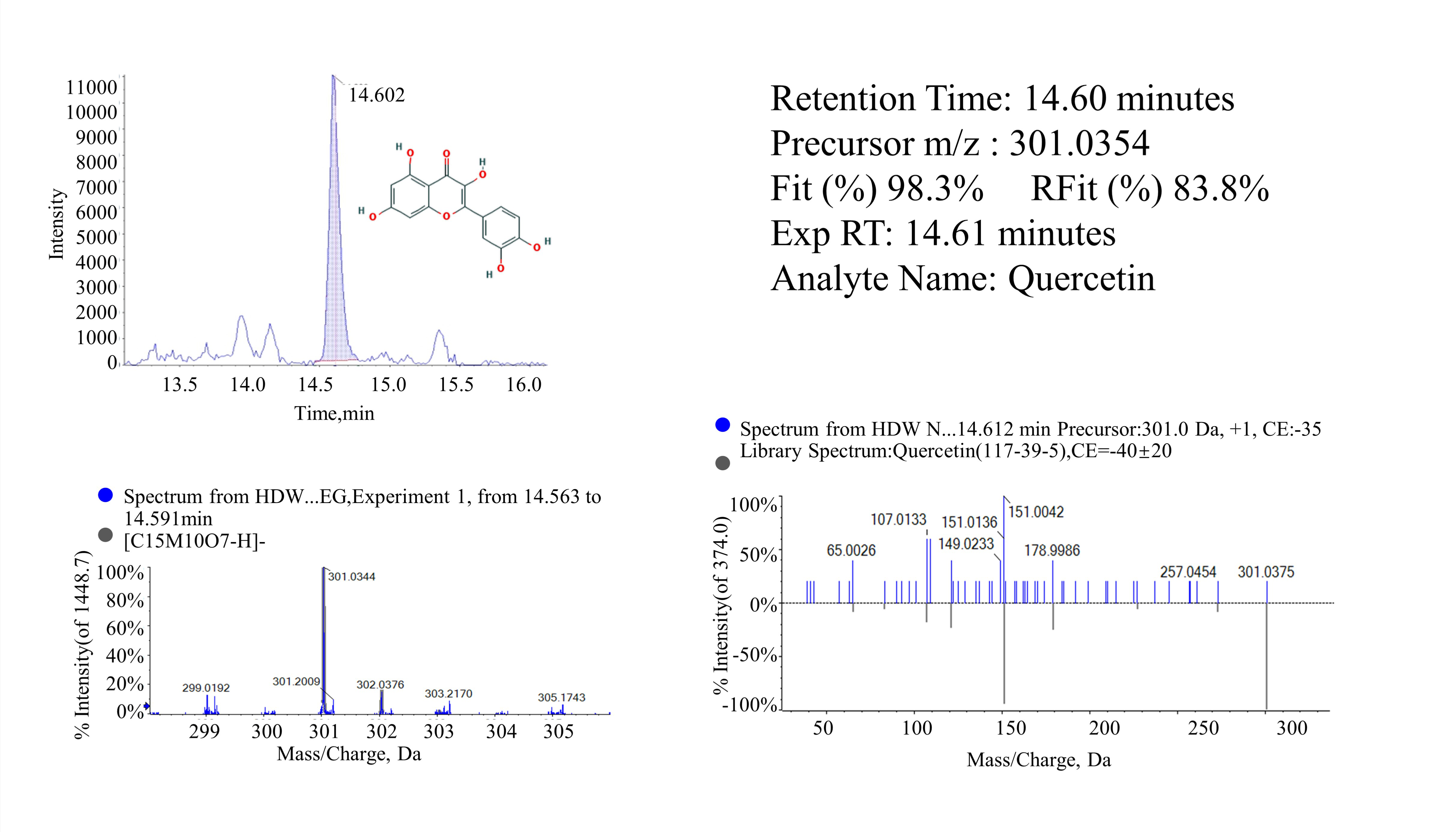


Supplymental figure 1a. Quercetin mass spectrometry identification


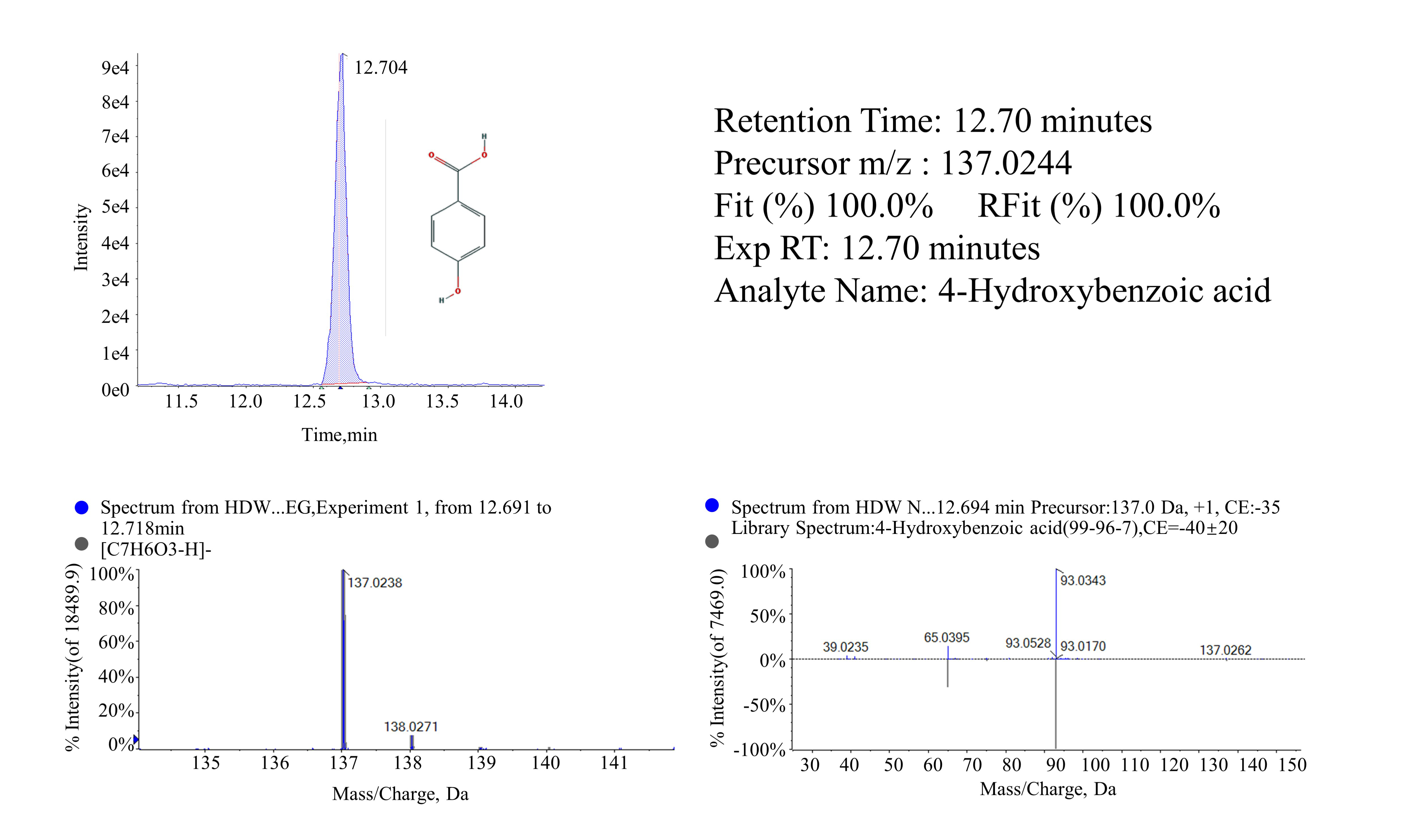


Supplymental figure 1b. 4-Hydroxybenzoic acid mass spectrometry identification


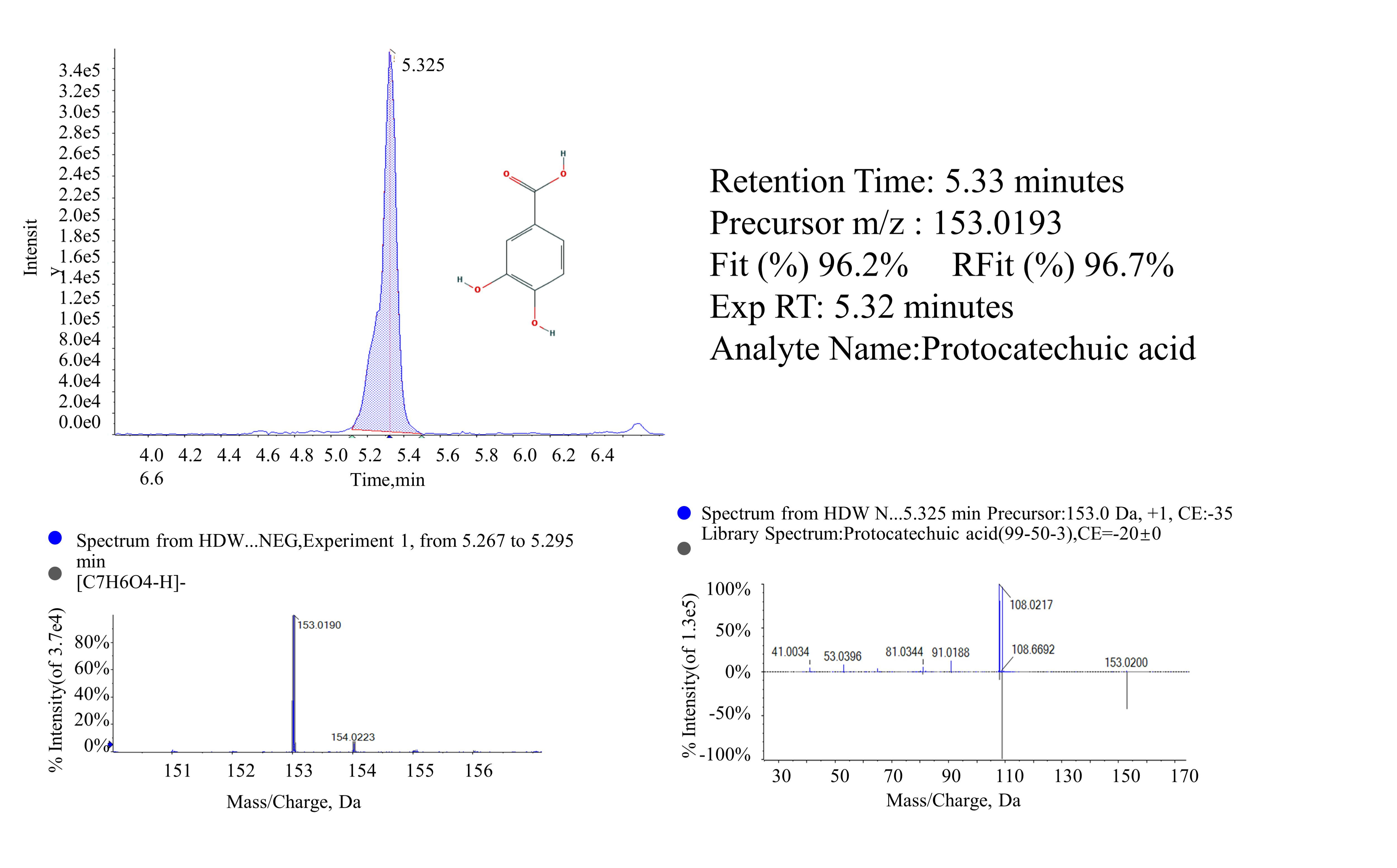


Supplymental figure 1c. Protocatechuic acid mass spectrometry identification


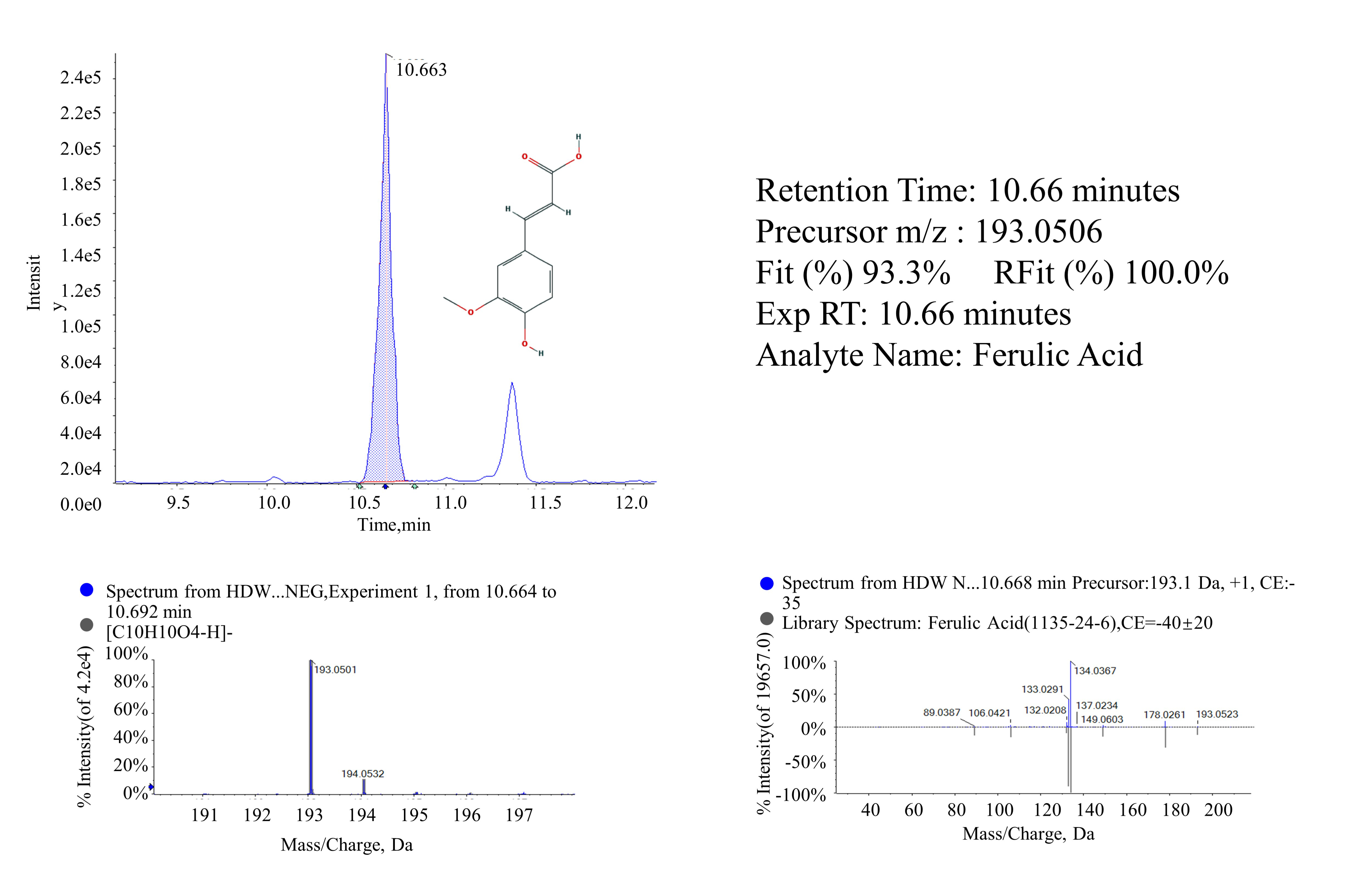


Supplymental figure 1d. Ferulic Acid mass spectrometry identification


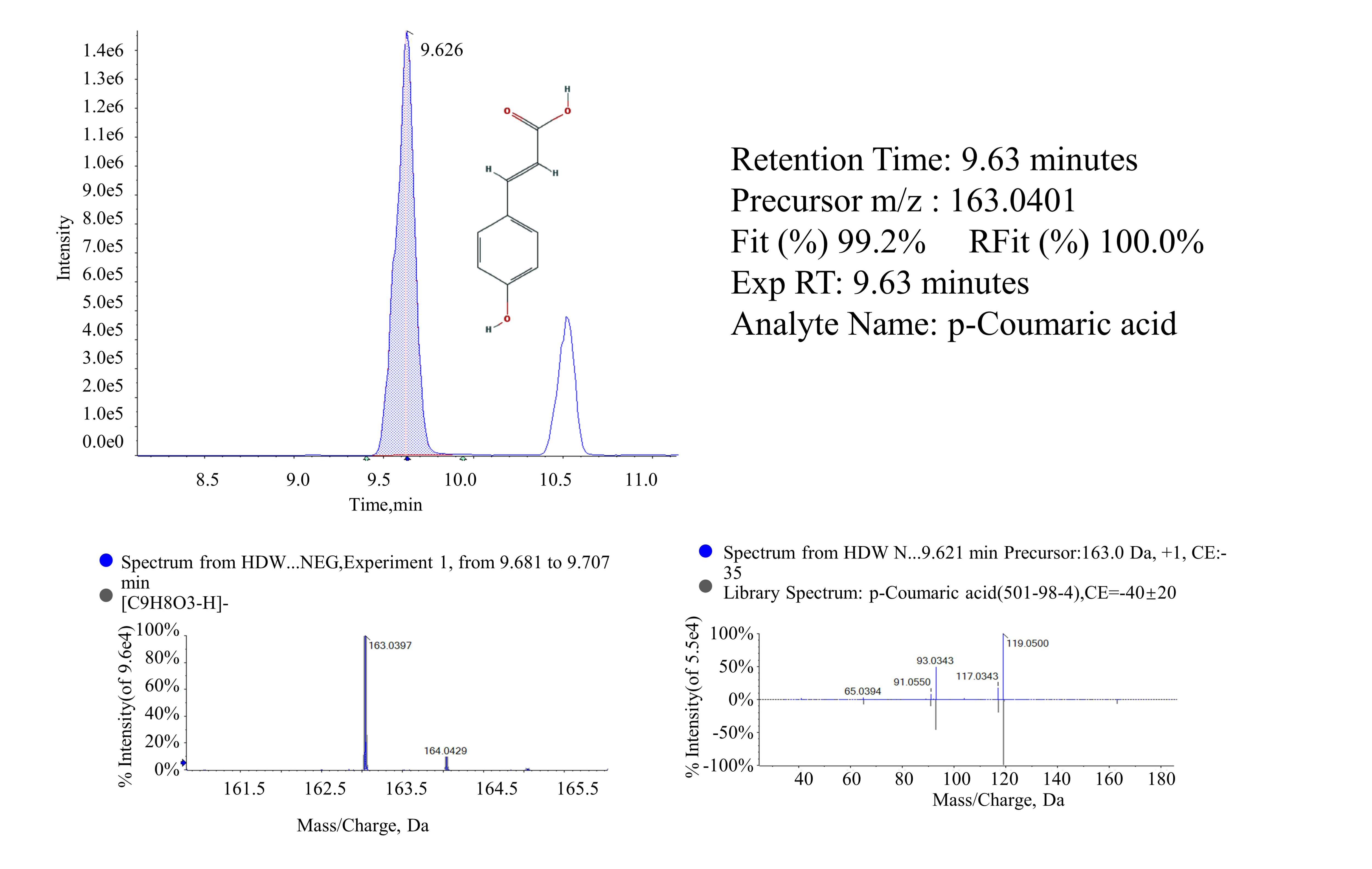


Supplymental figure 1e. P-Coumaric acid mass spectrometry identification


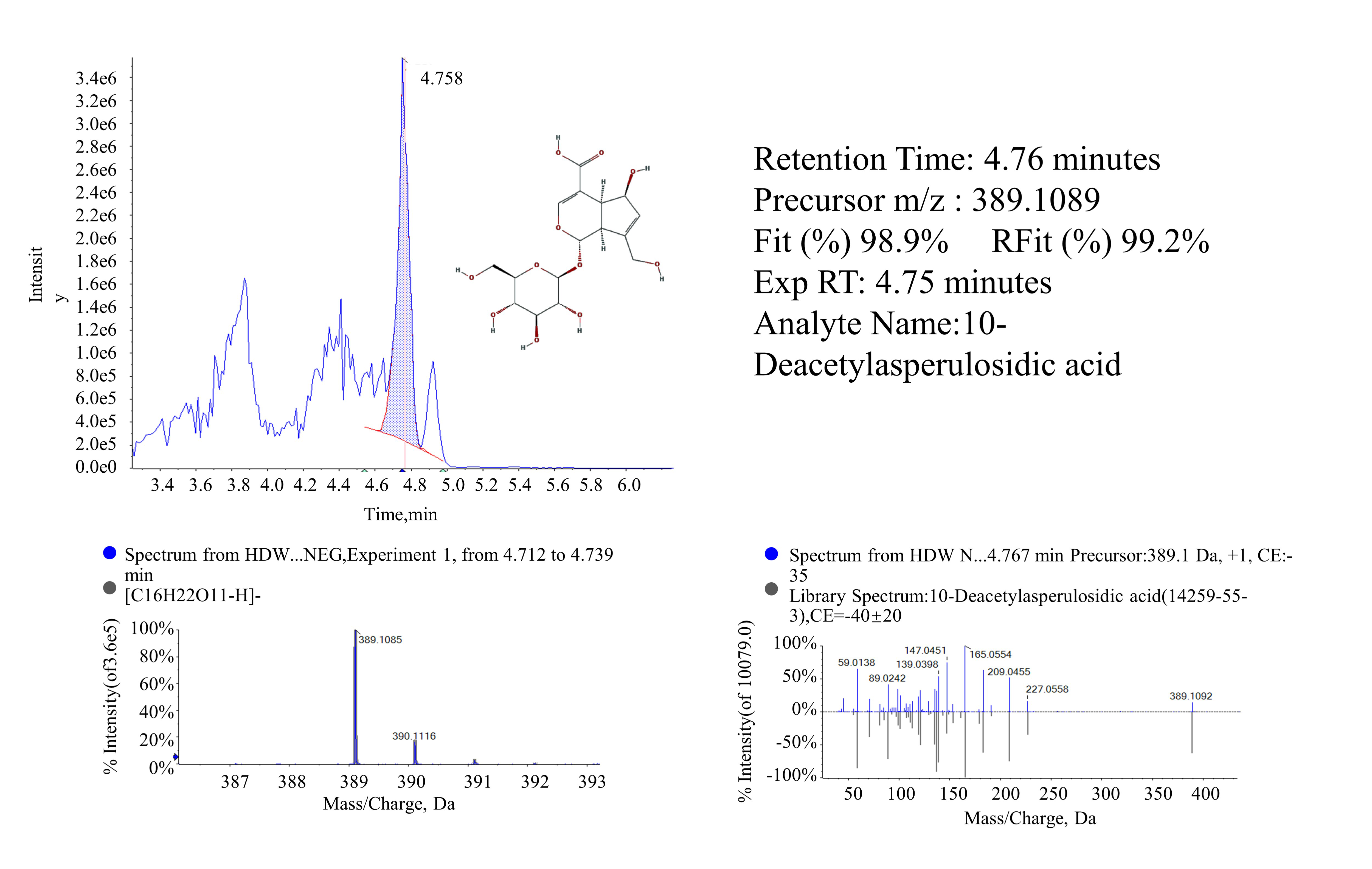


Supplymental figure 1f. 10-Deacetylasperulosidic acid mass spectrometry identification


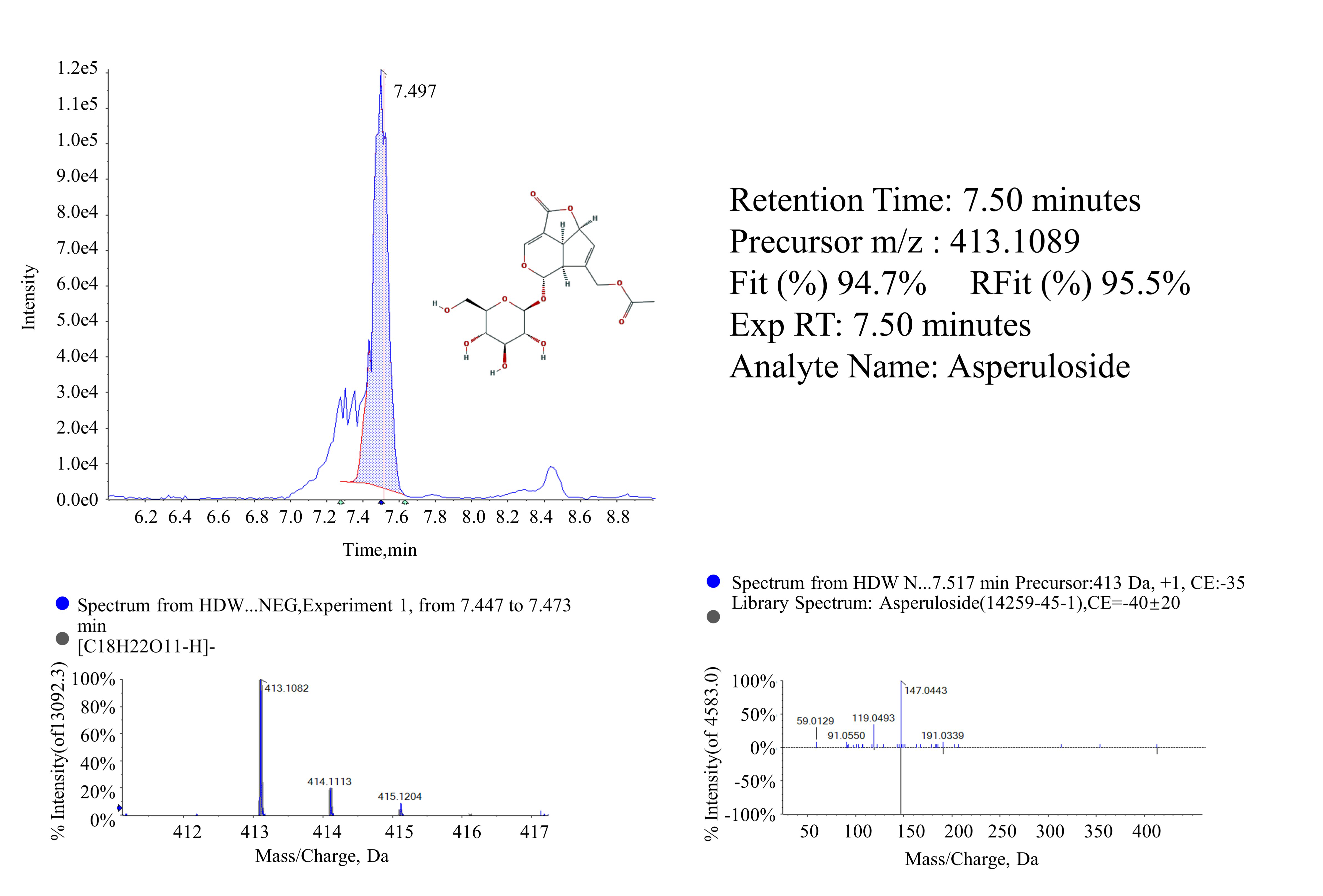


Supplymental figure 1g. Asperuloside mass spectrometry identification.

Supplymental table1. Ingredient information table

| NO. | Molecule ID | molecule_name | OB (%) | Drug-likeness |
| --- | --- | --- | --- | --- |
| 1 | MOL000098 | quercetin | 46.43 | 0.28 |
| 2 | MOL000103 | 4-Hydroxybenzoic acid | 30.15 | 0.03 |
| 3 | MOL000105 | protocatechuic acid | 25.37 | 0.04 |
| 4 | MOL000360 | ferulic acid | 39.56 | 0.06 |
| 5 | MOL000771 | p-coumaric acid | 43.29 | 0.04 |
| 6 | MOL001664 | deacetylasperulosidic acid | 3.42 | 0.45 |
| 7 | MOL007785 | asperuloside | 12.72 | 0.71 |
| 8 | MOL000114 | vanillic acid | 35.47 | 0.04 |
| 9 | MOL000422 | Kaempferol | 41.88 | 0.24 |
| 10 | MOL000511 | ursolic acid | 16.77 | 0.75 |
| 11 | MOL001649 | 2-hydroxy-3-methyl anthraquinone | 26.09 | 0.18 |
| 12 | MOL001668 | Geniposidic acid | 19.59 | 0.41 |
| 13 | MOL004474 | 4-vinylphenol | 48.44 | 0.02 |
| 14 | MOL009514 | 2-hydroxy-1-methoxy anthraquinone | 8.70 | 0.21 |
| 15 | MOL010592 | asperulosidic acid | 8.73 | 0.58 |


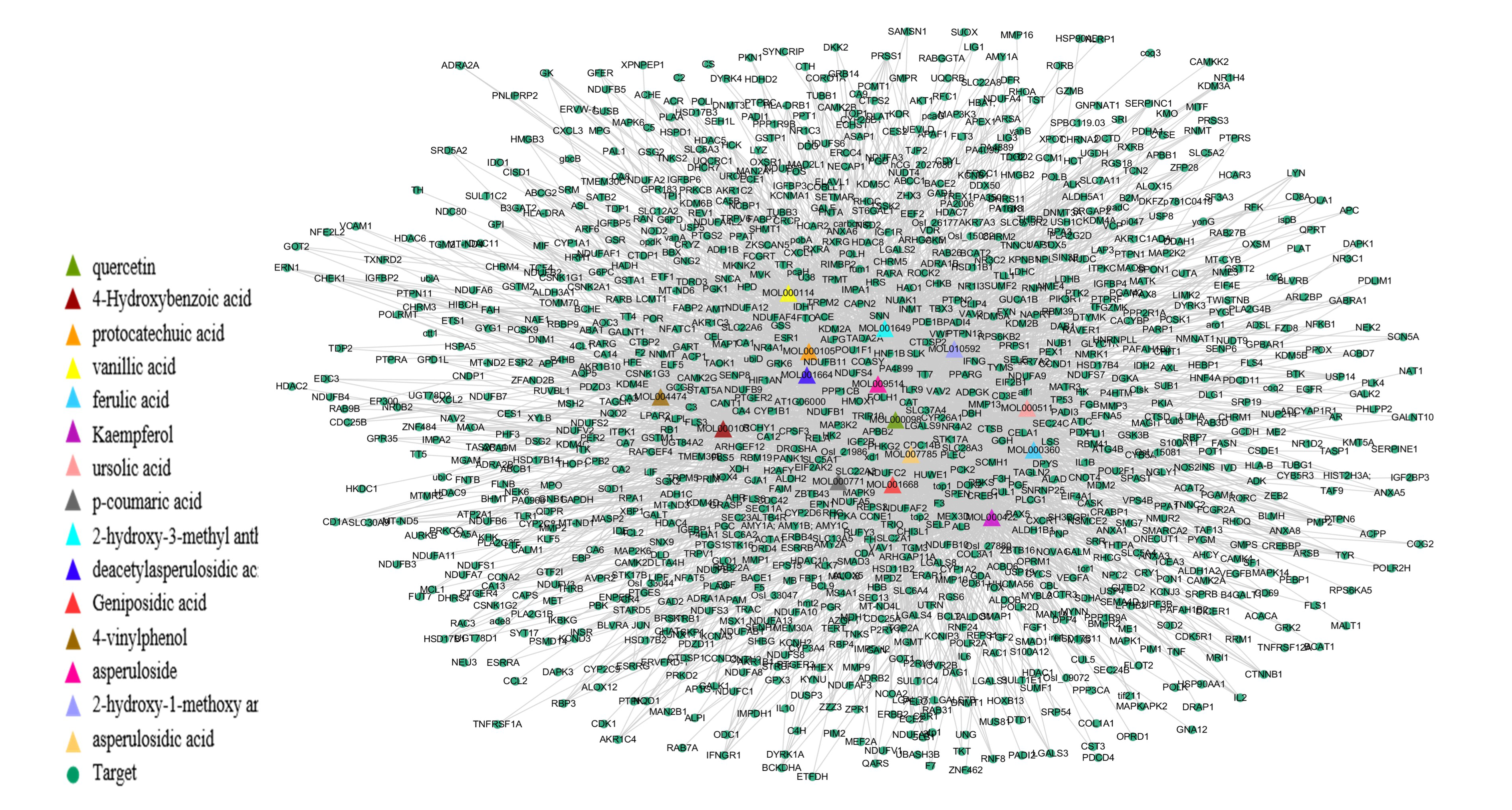


Supplymental Figure 2. Compound-target network diagram of *Hedyotis diffusa* Wild


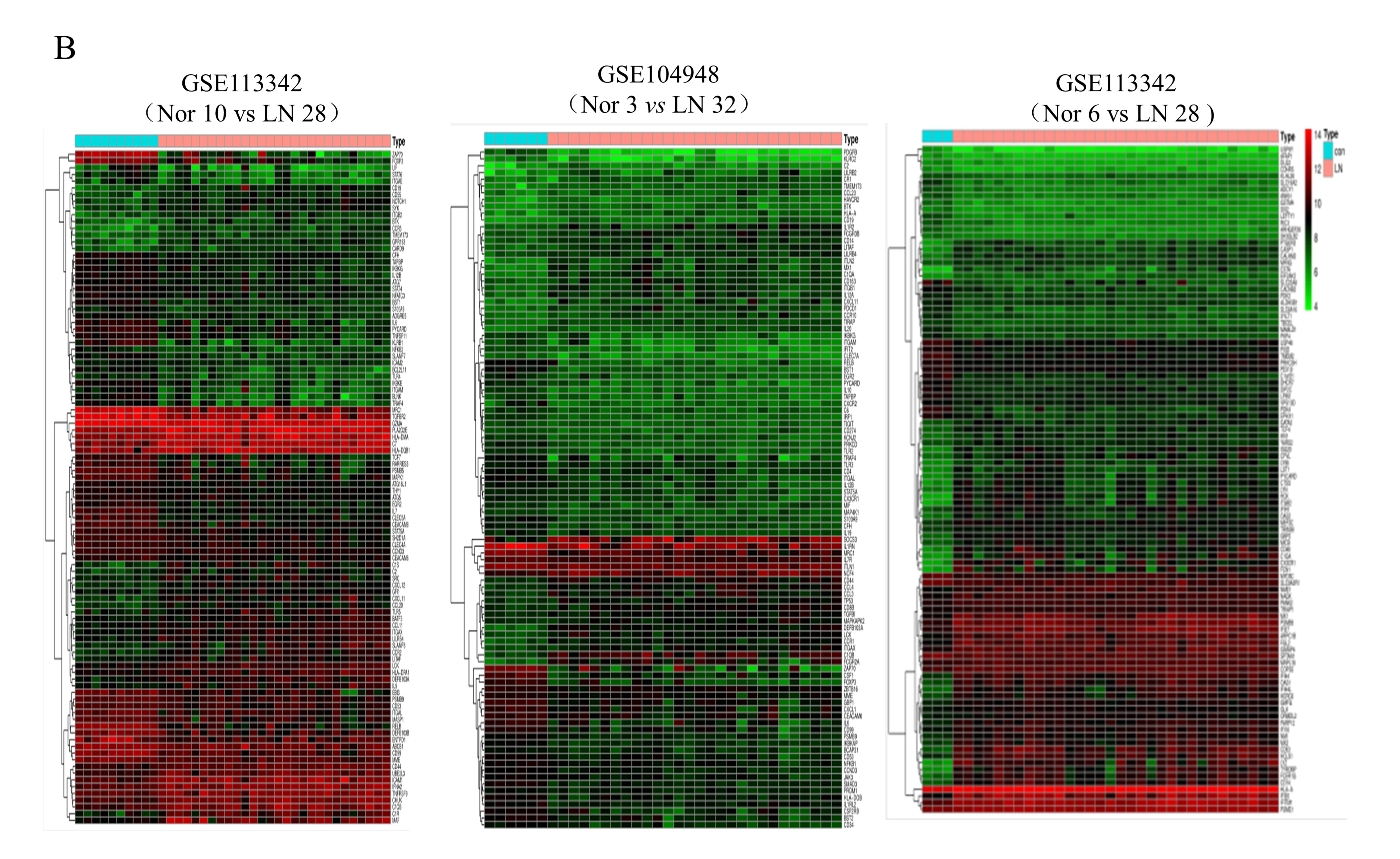


Supplymental Figure 3. Gene heat map. In the gene heat map, red and green represent upregulated and downregulated genes in the sample, respectively, whereas black represents no significant difference.
